# Common physiological responses during TTM

**DOI:** 10.1186/1471-227X-15-S1-A14

**Published:** 2015-06-24

**Authors:** Guillaume Geri, Benoit Champigneulle, Wulfran Bougouin, Michel Arnaout, Alain Cariou

**Affiliations:** 1 Medical Intensive Care Unit, AP-HP, Cochin Hospital, Paris, France; 2 Paris Descartes University – Medical School, Paris, France; 3 INSERM U970, Cardiovascular Research Center, European Georges Pompidou Hospital, Paris, France

## 

Achieving and maintaining perfect homeostasis, particularly in terms of metabolism, represent a major goal for post-cardiac arrest (CA) care. Since cooling may provoke different physiological responses, it is of particular importance to be aware of these changes that may require specific treatment adjustments during this recovery period [1]. Several points are particularly illustrative. Regarding ventilatory management, induced therapeutic hypothermia (TH) is known to decrease CO_2_ production, which may result in deleterious hypocapnia. Blood gases and expiratory tidal CO_2_ should be cautiously monitored in order to adapt ventilator settings since pronounced hypocapnia can provoke a decrease in cerebral blood flow that may alter brain perfusion [2]. Conversely, hypercapnia, leading to cerebrovascular vasodilatation and increased intracranial pressure, should also be banned. Regarding metabolic control, the correction of electrolyte and acid–base disturbances is essential and special attention should be given to those that may participate in the recurrence of CA or worsening of organ dysfunction (potassium, arterial pH). Concerning glycemia, consistent data underline that blood glucose variability seriously impairs the outcome of these patients rather than the mean level of glycemia, so attention should be paid to avoid such glycemia fluctuations [3]. Besides severe shock and brain injuries, patients with successfully resuscitated CA are also exposed to infectious complications, a supplementary insult which may affect a large number of survivors. Experimental hypothermia impairs immune functions and inhibits the secretion of proinflammatory cytokines and may suppress leukocyte migration and phagocytosis. In humans, TH is associated with an increased risk of early onset pneumonia [4]. Furthermore, the diagnosis of these infectious events is complicated in patients after CA, not only by the physiological effects of TH but also by the consequences of post-cardiac arrest syndrome. Even if they do not impact on survival or neurological outcome, these infectious events increase the duration of mechanical ventilation and hospital length of stay and should be managed using tailored preventive and therapeutic strategies. Finally, TH might also induce physiological changes in coagulation that may promote acute stent thrombosis. Experimental models showed that hypothermia may induce platelet activation, thrombus formation and stabilization, and several clinical studies reported an unexpected high rate of acute stent thrombosis in cardiac arrest patients after cooling for CA, which may compromise the benefit associated with early coronary reperfusion [5]. On the whole, being aware of all physiological changes induced by TH is crucial in order to maximize the benefit of this treatment in clinical practice.

## Financial disclosure

AC has received honorarium and/or travel costs from C. R. BARD and from Lilly France.

## Declaration

This abstract and supplement was proposed, developed and commissioned by BMC Emergency Medicine and was funded by an educational grant from C. R. BARD, NJ, USA. The published abstract was independently prepared by the author. C. R. BARD had no input into the content.
